# Supplementary material for: Identification of Different Putative Outer Membrane Electron Conduits Necessary for Fe(III) Citrate, Fe(III) Oxide, Mn(IV) Oxide, or Electrode Reduction by Geobacter sulfurreducens
Source: J Bacteriol. 2018 Sep 10;200(19):e00347-18. doi: 10.1128/JB.00347-18 (PMC6148476; doi:10.1128/JB.00347-18)
Supplement: Supplemental file 1 [file zjb999094880s1.pdf]

Supplemental Figures

Figure S1

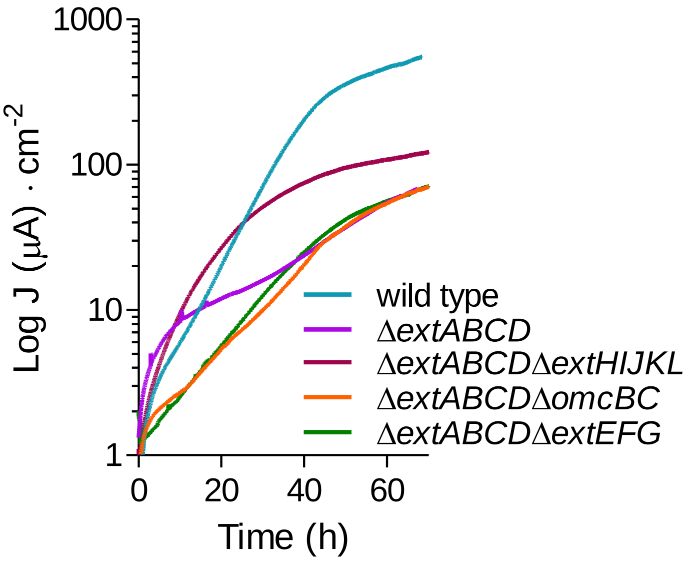

Figure S1. Deletion of second clusters in Δ*extABCD* background results in residual activity. Deletion of *extEFG*, *extHIJKL*, and *omcBC* in a Δ*extABCD* strain did not result in additional defects in electrode reduction. Subsequent deletions resulted in the "*extABCD*$^{+}$" strain shown in Fig 2B.

Figure S2

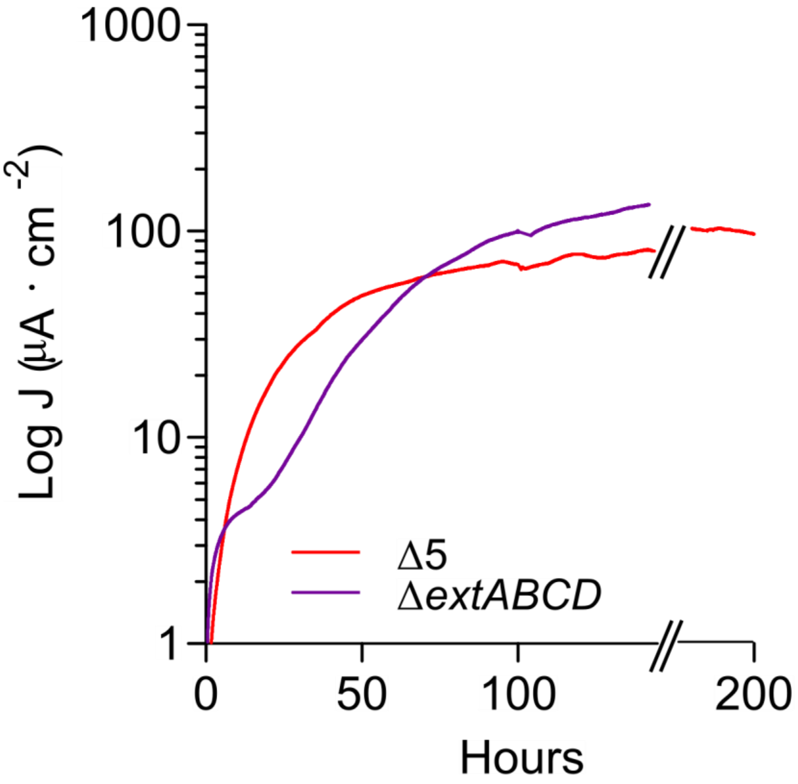

Figure S2. Δ*extABCD* and Δ5 suppressor strains do not arise after 150 hours. Incubation of Δ*extABCD* and Δ5 on electrodes poised at +240 mV for more than 150 hours did not result in a change in phenotype that would be indicative of suppressor strains. The Δ5 strain was grown for 200 hours and this strain was still unable to produce more than 150 μA/cm^2. Curves representative of n = 4.

Table S1

Primers used in this study	
Function and primer ID	Sequence (5'-3')
Complementation vectors	
GSU2645 f AscI	ACGTCG GGCGCGCC CGG CCA TTT CAT TGC TTG ACA GG
GSU2645 -44p r o/l	CAATGCATCCCCCTCCTCGTG TCA GCG CTG ACG AAC CGG
GSU2644 –p f o/l	CCGGTTCGTCAGCGCTGA CAC GAG GAG GGG GAT GCA TTG
GSU2642 r BglII	ACGTCG AGATCT GCA GGC GGC GTC AAC GAA C
GSU2739 f NdeI	ACGTCG CATATG GGA ATC AAA GGG TTC ACT CGA C
GSU2737 r NheI	ACGTCG GCTAGC GTC CGC TAT TAC GGA CGG GT
Confirmation of gene deletion (additional to those described in Chan *et al.*, 2017)	
GSU2737 or GSU2731 f NdeI	ACGTCG CATATG GGA GGA AAT GGC ATG AGT AGA AAA G
GSU2737 r2 NheI	ACGTCG GCTAGC CTA TTA CGG ACG GGT CGT GCC

Table S2

RNAseq raw reads (see Excel file)

Table S3

Detailed list of conduit cluster homolog locus tags (see Excel file)
